# Asap1 Affects the Susceptibility of Zebrafish to *Mycobacterium* by Regulating Macrophage Migration

**DOI:** 10.3389/fcimb.2020.519503

**Published:** 2020-10-29

**Authors:** Jia Cui, Guangxin Chen, Da Wen, Yuhuan Wang, Zhonghua Zhao, Changxin Wu

**Affiliations:** ^1^Institutes of Biomedical Sciences, Shanxi University, Taiyuan, China; ^2^Department of Microbiology, Changzhi Medical College, Changzhi, China; ^3^Shanxi Academy of Advanced Research and Innovation, Taiyuan, China

**Keywords:** ASAP1, *Mycobacterium*, migration, zebrafish, macrophage

## Abstract

The ADP ribosylation factor (ARF) GTPase activation protein ASAP1 possesses multiple biological functions, including regulation of cytoskeletal dynamics, small GTP-binding protein receptor recycling, and intracellular vesicle trafficking. Recently, *ASAP1* polymorphisms have been reported to be associated with human susceptibility to tuberculosis (TB) according to a large-scale genome-wide association study (GWAS); ASAP1 expression affects dendritic cell migration, which may be involved in TB predisposition. However, it remains unclear whether ASAP1 affects TB *in vivo*. To address this issue, we used zebrafish as a model system to examine the effects of Asap1 against *Mycobacterium marinum*, an organism closely related to *Mycobacterium tuberculosis*. Two zebrafish *asap1* homologs (*asap1a and asap1b*) were identified and characterized. By morpholino knockdown of *asap1a* and *asap1b* as a whole, we found that the *asap1* morphants showed a higher mycobacterial load than the controls, which was almost rescued by injecting *asap1* mRNA that confers resistance to mycobacterial infection. These Asap1-depleted zebrafish also exhibited decreased macrophage migration in response to tail injury or upon infection with *M. marinum* in the hindbrain ventricle, which was also proved in THP1-derived macrophages of knockdown ASAP1. Together, these findings represent a new perspective on the role of Asap1 in resistance to mycobacterial infection.

## Introduction

Despite advances in diagnosis and treatment, tuberculosis (TB) remains a major global health concern, causing millions of deaths annually (Lewinsohn and Lewinsohn, 2019). It has been estimated that approximately one-third of the world's population has been infected with the pathogen *Mycobacterium tuberculosis* (*Mtb*), with nearly 5–10% of infected individuals progressing into active TB, although most remain in a latent state (Marino et al., 2004; Nguyen-Chi et al., 2015). The World Health Organization (WHO) set a goal to end TB by 2030, but with the emergence of antibiotic-resistant or multi-antibiotic-resistant strains, this is a huge challenge (WHO, 2019). Besides, as the specific pathogenesis of TB is still unclear, it is difficult to effectively treat patients with TB to completely eradicate the infection.

ASAP1 (ArfGAP with SH3 domain, ankyrin repeat, and PH domain 1), a member of the ArfGAP (ArfGTPase-activating protein) family, plays a major regulatory role in cell membrane remodeling, cytoskeletal organization, and tumor invasion and metastasis (Randazzo et al., 2000, 2007; Liu et al., 2002; Tien et al., 2014; Schreiber et al., 2019). Recently, the function of ASAP1 has also been explored in the field of infectious diseases, especially with regard to TB susceptibility. A recent genome-wide association study (GWAS) reported that single-nucleotide polymorphisms in the *ASAP1* intron were associated with the risk of TB in a Russian population (Curtis et al., 2015). Subsequently, two genetic polymorphism sites in *ASAP1* were also found to be related to TB susceptibility in a Chinese Xinjiang Muslim population (Wang et al., 2018). Moreover, an allele of rs4733781 in the *ASAP1* intron has been associated with a decreased occurrence of TB in the Han Chinese population (Chen et al., 2019). Nevertheless, the detailed regulatory effects of ASAP1 during host infection with *Mycobacterium* remain to be elucidated.

The interaction between the host immune system and *Mycobacterium* is very complex, with the most focused site of interplay being the tuberculosis granuloma, an organized structure consisting of macrophages, T and B lymphocytes, and various other immune cells (Davis and Ramakrishnan, 2009; Silva Miranda et al., 2012; Huang et al., 2017). Among these cells, macrophages serve as important phagocytic and primary constituent cells, and play a significant role in the host innate immune response against mycobacterial infection, including from the primary infection to bacillary dissemination, and from latency to activation of TB (Das et al., 2013; Korb et al., 2016; Nathan, 2016). Notably, a recent study showed that the loss of ASAP1 impaired dendritic cell migration, which may affect the host adaptive immune response to TB infection (Curtis et al., 2015). However, the effect of ASAP1 expression level on macrophage migration has not been reported.

Progress toward fully understanding the occurrence and development of TB has also been hindered by the lack of an appropriate and tractable infectious animal model that can suitably account for the complexity of host genetic factors and pathogen interactions (Guirado et al., 2015). As an alternative, the model of zebrafish infected with *M. marinum* (*Mm*), a close genetic relative of *Mtb*, has proven very effective for understanding TB pathogenesis, given the ability of *Mm* to induce granuloma-like disease (Helguera-Repetto et al., 2004; Cronan and Tobin, 2014). This model also allows the evaluation of host innate immune reaction during the first week of life, as the zebrafish adaptive immunity is not yet mature at the early larval developmental stage (Tobin and Ramakrishnan, 2008; van der Vaart et al., 2013; Tien et al., 2014; Moule and Cirillo, 2020)

In this study, we explored the role of ASAP1 in macrophage migration during *Mtb* infection, taking advantage of the optical transparency of the zebrafish larvae infection model. In particular, we explored the function of Asap1 in the process of zebrafish infection with *Mm* within the first 10 days of development, when host and pathogen interact in the sole context of innate immunity *in vivo*. These analyses will expand current understanding on the role of ASAP1 in anti-mycobacterial infection, and explore a preliminary mechanism for ASAP1-mediated TB susceptibility.

## Materials and Methods

### Bacterial Culture

The pTEC27 plasmid (Takaki et al., 2013) expressing the tdTomato protein, a kind gift from Lalita Ramakrishnan (Department of Medicine, University of Cambridge, UK), was used to transform *Mm* by electroporation. *Mm* was a kind gift from Professor Chen Niu (School of Basic Medical Sciences, Fudan University, China). tdTomato-*Mm* were cultured at 28°C in 7H9 broth supplemented with 0.2% glycerol, 10% albumin–dextrose–catalase (BINDER, Qingdao, China), 0.05% Tween 80, and hygromycin (50 ng/mL) or kanamycin (50 ng/mL). *Mtb* strain H37Ra was a kind gift from Professor Yujiong Wang (School of Life Science, Ningxia University, China) and cultured in 7H9 broth with the same enrichment albeit at 37°C. All bacteria were washed with phosphate buffered saline (PBS), and bacterial clumps were destroyed by passing through syringes prior to use for injecting into zebrafish or infecting macrophages.

### Zebrafish Lines and Ethics Statement

All zebrafish experiments were carried out according to the China guidelines for the handling of laboratory animals. The ethics committee of Shanxi University approved this study (SXULL2019004). Wild-type zebrafish (AB) were purchased from the China Zebrafish Resource Center (CZRC) and maintained in a recirculating aquatic system at a constant temperature (28°C) with a 10 h dark/14 h light cycle.

### Quantitative Reverse Transcription-Polymerase Chain Reaction (qRT-PCR) Analysis

Total RNA of zebrafish embryos or larvae at different developmental stages were isolated using TRIzol reagent (TaKaRa, Shiga, Japan) according to the manufacturer's protocol, and 1,000 ng RNA was used for reverse transcription to cDNA using the PrimeScript™RT Master Mix kit (TaKaRa). qRT-PCR was performed on a LightCycler 480 system (Roche, Basel, Switzerland) using SYBR-Green mix (Bio-Rad, Hercules, CA, USA) under the following conditions: initial denaturation for 5 min at 95°C, 45 cycles of amplification including denaturation step: 15 s at 95°C, annealing stage: 45 s at 58°C, and extension step: 1 min at 72°C. PCR amplification of the zebrafish β-actin gene was concomitantly performed as a reference. Data were normalized to β-actin using the 2^−ΔΔCt^ method. The primers used are shown in Table 1.

**Table 1:** Primer sequence used in this study.

**Primer name**	**Sequence (5^**′**^-3^**′**^)**	**Application**
*asap1a*-probe-F	AGCAAAGCACTACGTCTATGAAC	*asap1a* probe plasmid
*asap1a*-probe-R	GTCTGACAGAATGTGCACGAAG	
*asap1b*-probe-F	TGGCAGCTCTACCCTGTCAAAG	*asap1b* probe plasmid
*asap1b*-probe-R	CTGGTTTAGGTGGTAATTCTGAAGG	
*asap1a*-q -F	AATGTTCTGGAAGAGGCTTTGGA	*asap1a* qPCR
*asap1a*-q -R	GTTATCTCGGCTGATGAAGTTGC	
*asap1b*-q-F	CCAACCTCCAGATACTCCAACAA	*asap1b* qPCR
*asap1b*-q-R	GGCAATCATAAATAGTCTTCACCCT	
β-actin-q-F	CTCTTCCAGCCTTCCTTCCT	β-actin qPCR
β-actin-q-R	CACCGATCCAGACGGAGTAT	
*asap1a*-F	ATGAGGTCCTCGTCCTCGCGTTTG	
*asap1a*-R	TCAGTCTGACAGAATGTGCACGAA	*asap1a* clone
*asap1b*-F	ATGAGGTCATCATCCTCTCGTCTTAG	
*asap1b*-R	TCAATCTGATAAGATATGAACAAA	*asap1b* clone

### Whole Mount *in situ* Hybridization

*asap1a* and *asap1b* probe template genes were amplified from 3 days post-fertilization (dpf) zebrafish by reverse transcription-polymerase chain reaction (RT-PCR) using the probe primers (Table 1) and ligated with pGEM-T vectors. Digoxigenin (DIG)-labeled sense and anti-sense RNA probes were transcribed according to the kit instructions (Promega, Madison, WI, USA) from recombinant pGEM-T-*asap1a* and pGEM-T-*asap1b* probe plasmids. The sense probe served as negative control. The detailed process of *in situ* hybridizations on whole mount embryos was previously described (Nguyen-Chi et al., 2015; Sanderson et al., 2015). Briefly, embryos from different periods were collected and the chorions were gently removed by hand. In particular, 0.0045% 1-phenyl-2-thiourea (PTU; Sigma Aldrich, St. Louis, MO, USA) solution was used to prevent the formation of melanin pigment in 24 h post-fertilization (hpf) embryos. Next, dechorionated embryos were fixed in 4% paraformaldehyde in PBS overnight at 4°C and dehydrated in 100% methanol for at least 2 h at −20°C. Then, the embryos were subjected to rehydration, prehybridization, and hybridization with antisense DIG-labeled RNA (Roche), blocked for unspecific binding sites in 1% bovine serum albumin (Solarbio, Beijing, China) and 1% sheep serum (Solarbio), and incubated with anti-DIG antibody (1:10,000; Roche). Finally, anti-DIG alkaline phosphatase (Roche) and chromogenic substrate NBT/BCIP (Roche) were used to stain the embryos. Images were obtained using light microscopy (SZX16, Olympus, Tokyo, Japan) with a 6.3 × objective.

### Morpholino

Morpholino oligonucleotide (MO) (Gene Tools, Philomath, OR, USA) were diluted to 0.5 mM in 1 × Danieau buffer [58 mM NaCl, 0.7 mM KCl, 0.4 mM MgSO_4_, 0.6 mM Ca (NO_3_)_2_, and 5.0 mM HEPES; pH 7.6] containing 10% phenol red (Sigma Aldrich). 1 nL MO or MO mixture (MOs) were injected into 1-cell stage embryos using an injector (PV830; WPI, Sarasota, FL, USA) as described (Clay et al., 2008; Yuan and Sun, 2009; Roca and Ramakrishnan, 2013). *asap1a* (ENSDART00000144870.3) ATG-MO (5′-GAG GAC CTC AAG GCT CTG TAG TCA C-3′) plus *asap1b* (ENSDART00000145466.3) ATG-MO (5′-TAA GAC GAG AGG ATG ATG AC-3′) were used as the MO-*asap1* mixture, *asap1a* control MO (5′-GAG CAG CTC ATC GCT CTC TAC TCA C-3′) plus *asap1b* control MO (5′-TAA CAC CAG ACG ATC ATC ACC TCA T-3′) were used as the MO-NC mixture.

### Western Blotting of Zebrafish

Approximately 100 embryos or larvae at each stage of 1, 5, and 10 dpf were prepared for protein extraction. One dpf embryos were particularly removed from chorions in batches by using 1 mg/mL pronase (Sigma Aldrich) and swirling occasionally at 28°C for 17 min until complete dechorionation. The floating chorions were decanted after rinsing three times in cold Dulbecco's PBS (DPBS) solution. All the dechorionated embryos or larvae were gently blown repeatedly in DPBS solution and lightly centrifugated at 24 × g for 10 min at 4°C, the supernatant (yolk) was removed. Dechorionated and deyolked embryos or larvae were transferred to cold lysis buffer [20 mM Tris-HCl, pH 7.4, 10% glycerol, 187 mM NaCl, 2 mM ethylenediaminetetraacetic acid, 1% Triton X-100, and protein inhibitor cocktail (Sigma Aldrich)], lysed on ice for 30 min and pipetted every 10 min, followed by centrifugation at 10,000 × g for 10 min at 4°C. In particular, 5 and 10 dpf larvae were homogenized with ultrasonic disruption at 500 W until uniform in consistency and then centrifugated. The supernatant (zebrafish protein) was collected, resolved by 10% sodium dodecyl sulfate-polyacrylamide gel electrophoresis, transferred to immunoblot polyvinylidene difluoride membranes (Millipore, Billerica, MA, USA). The membrane was blocked using 5% non-fat milk with 0.5% Tween-20 for 1 h at room temperature and washed three times with Tris-Buffered Saline Tween-20 (each 10 min). The membrane was then incubated using the primary antibody against ASAP1 with a peptide from mouse as immunogen (1:2,500; Abcam11011, Cambridge, UK) and β-actin (1:5,000; Proteintech, Rosemont, IL, USA) at room temperature for 1 h, and then washed three times with Tris-Buffered Saline Tween-20. Next, the membrane was incubated using horseradish peroxidase-labeled secondary goat anti-rabbit (1:2,000; absin, Shanghai, China) or goat anti-mouse (1:2,000; absin) antibody for 1 h at room temperature, followed by three washes with Tris-Buffered Saline Tween-20. The membrane was visualized using a commercial ECL kit (GE Healthcare, Chicago, IL, USA).

### Injection of *Mm* Into Zebrafish

Embryos were collected and kept in E3 medium (5 mM NaCl, 0.17 mM KCl, 0.33 mM CaCl_2_, 0.33 mM MgSO_4_, 10% methylene blue). tdTomato-*Mm* were prepared and 50 colony-forming units (CFU) microinjected into 16–1,000 cell stage or 100 CFU into the caudal vein of 28–30 hpf embryos according to published procedures (Takaki et al., 2013). Previously, PTU was added to the E3 medium to prevent melanization when the embryos were ~24 hpf. Subsequently, confocal images (LSM710, Carl Zeiss, Germany) were acquired from zebrafish larvae at 5 days post- infection (dpi) under anesthesia with 0.016% ethyl 3-aminobenzoate (Tricaine, Sigma Aldrich) in 2.5% methyl cellulose (Sigma Aldrich) drop. Finally, the overall bacterial burden of whole larvae was quantified by fluorescence quantification of the images using image J software (National Institutes of Health, Bethesda, MD, USA).

### Phenotype Rescue Experiments in Zebrafish

The full-length coding gene sequence of *asap1a* or *asap1b* were amplified by using specific primers (Table 1) from the cDNA of 3 dpf zebrafish and subcloned into pCS2+ vectors using the Gibson Assembly enzyme-reagent mixture (NEB, Frankfurt am Main, Germany). The recombinant pCS2+-*asap1a* or pCS2+-*asap1b* plasmids were linearized using the *Not* I restriction enzyme (NEB). *asap1a* or *asap1b* mRNA were prepared by *in vitro* transcription using mMESSAGE mMACHINE™ SP6 Transcription Kit (Ambion, Austin, TX, USA) from pCS2+-*asap1a* or *asap1b* plasmids. The synthetic mRNA, at the concentration of 200 ng/μL, were co-injected into 1-cell stage embryos with *asap1* morpholino mixture for rescue experiments according to the previous method (Rosen et al., 2009).

### Zebrafish Macrophages Recruitment Assay

Neutral red staining was used to observe the macrophage, following published procedures (Herbomel et al., 2001; Davis and Ramakrishnan, 2009; Shiau et al., 2015). The 3 dpf zebrafish were wounded consistently by tailfin transection with a sterile scalpel. Subsequently, zebrafish were immersed in neutral red solution (2.5 μg/mL + 0.003% PTU) (Sigma Aldrich) at 28°C in the dark for 7–9 h. Furthermore, in order to observe the response of macrophages against mycobacterial infection, *Mm* were injected into the hindbrain of 30–32 hpf zebrafish embryos with 50 CFU according to the previous method (Gutzman and Sive, 2009). Zebrafish were then incubated in neutral red solution as already described. Anesthetized zebrafish were embedded into solidified agarose drops and viewed under light microscopy to acquire images (SteREO Discovery.V20; Carl Zeiss, Oberkochen, Germany).

### siRNA Transfection

Human monocyte-like THP-1 cells were purchased from the Type Culture Collection of the Chinese Academy of Sciences (Shanghai, China) and cultured in RPMI-1640 supplemented with 10% fetal bovine serum (Gibco, Grand Island, NY, USA). A pool of three ASAP1-specific small-interfering RNA (siRNA) or a control siRNA (GenePharma, Shanghai, China) (ASAP1 siRNA: 5′-CAC CUU GGA UUC UUU GUU A-3′, 5′-GAC CUG AUA UCA CAU AAU A-3′, 5′-CUG CCC UAG ACA UAG CAA A-3′; control siRNA 5′-CAU GCA UCG UAG CUA UGC AUU-3′) was transfected into human monocyte-like THP-1 cells using Lipofectamine3000 (Thermo Fisher Scientific) following treatment using phorbol-12-myristate-13-acetate (PMA, Sigma Aldrich) at 100 ng/mL for 48 h. Total protein of 24 h post transfection was extracted from the THP-1 cells using lysis buffer (Solarbio). Western blotting was performed using an anti-ASAP1 antibody (1:1,000; sc374410, Santa Cruz Biotechnology, Dallas, TX, USA) and anti-β-actin antibody (1:5,000; Proteintech) to assess knockdown efficiency.

### Transwell Migration Assay

THP-1 cells were infected with recombinant strains tdTomato-H37Ra according to a previously described protocol (Behar et al., 2011). Firstly, THP-1 cells were seeded at a density of 10^6^ cells per well in RPMI-1640 medium supplemented with 10% fetal bovine serum for 48 h with PMA incubation and then infected with H37Ra at multiplicity of infection (MOI) of 5. After 12 h, the supernatant was collected for use as a chemoattractant in the subsequent Transwell assay. The cell migration assay was conducted in 24-well Transwell plates of 8.0-μm pore size (Corning Costar, Armonk, NY, USA), referring to the previous method (Smith et al., 2015). Prior to the assay, THP-1 cells with PMA incubation were starved overnight in RPMI-1640, and 2 × 10^4^ cells suspended in serum-free RPMI-1640 were seeded onto each upper chamber, whereas 10% FBS RPMI-1640 medium and the supernatant obtained following cell infection with H37Ra were mixed in equal volumes to 1 mL, and added to the lower chambers. The Transwell plates were maintained at 37°C in a humidified atmosphere with 5% CO_2_. After 24 h, cells remaining on the upper surface of the membrane were completely removed with cotton swabs, whereas cells that migrated through the permeable membrane to the lower surface were fixed in 4% paraformaldehyde followed by staining with 0.1% crystal violet (Solarbio). Images of migrated cells were acquired using light microscopy (DMi1, Leica, Wetzlar, Germany).

### Statistics

Results are presented as the mean ± SEM. All data were analyzed using Prism 7 software (GraphPad, La Jolla, CA, USA). Statistical significances were evaluated by the Kruskal-Wallis and the Dunn post-test among groups, the Mann-Whitney test or the Student's unpaired *t*-test between two groups. The significant value was set at *P* < 0.05.

## Results

### Characterization of the Zebrafish *asap1* Gene

**Figure 1:**
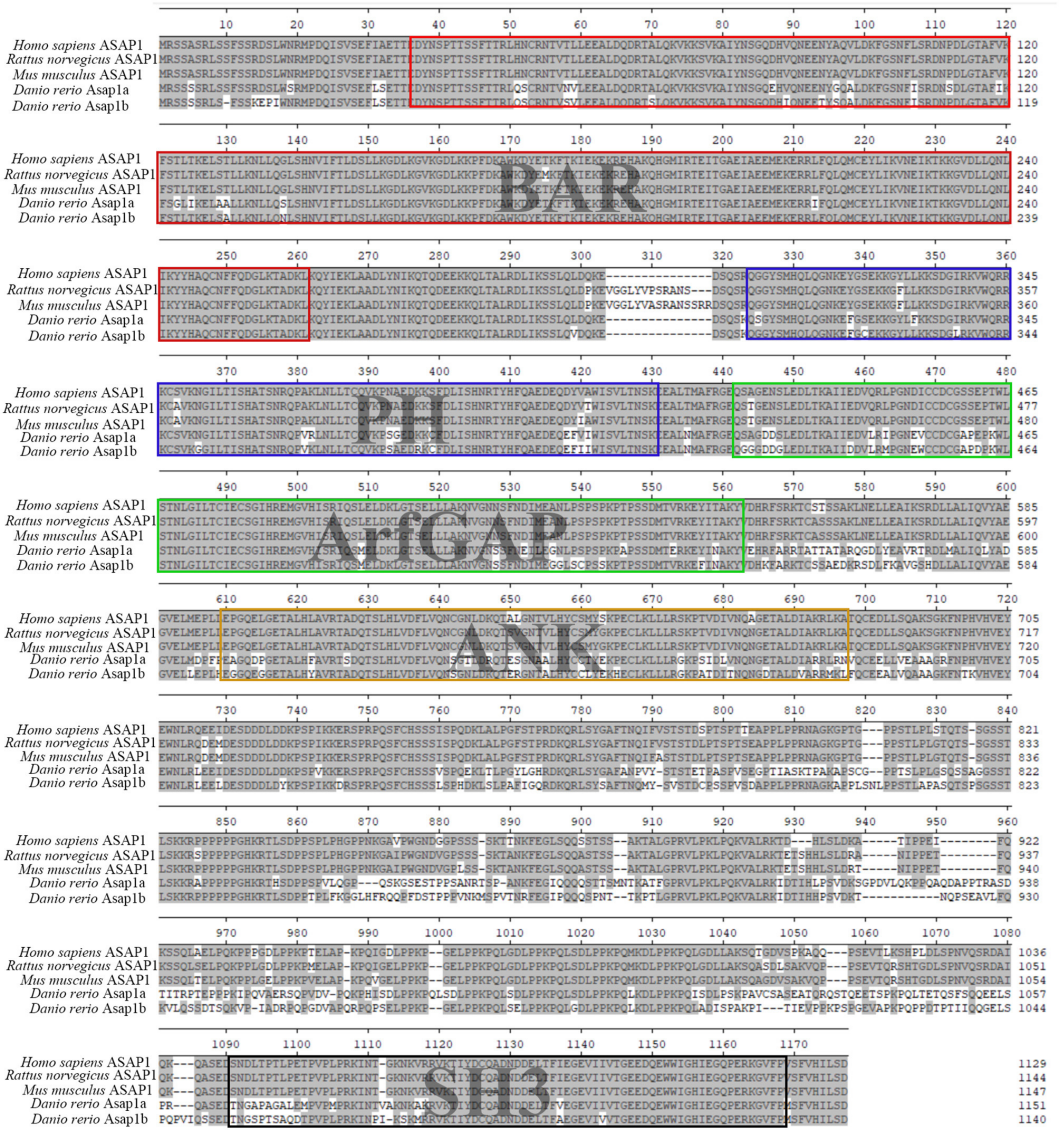
Multiple sequence alignment of zebrafish Asap1a (ENSDART00000144870.3) and Asap1b (ENSDART00000145466.3) with human ASAP1 (ENST00000518721.6), rat ASAP1 (ENSRNOT00000079524.1), and mouse ASAP1 (ENSMUST00000177374.7). Five domains common between all ASAP1 are indicated by different color boxes.

**Figure 2:**
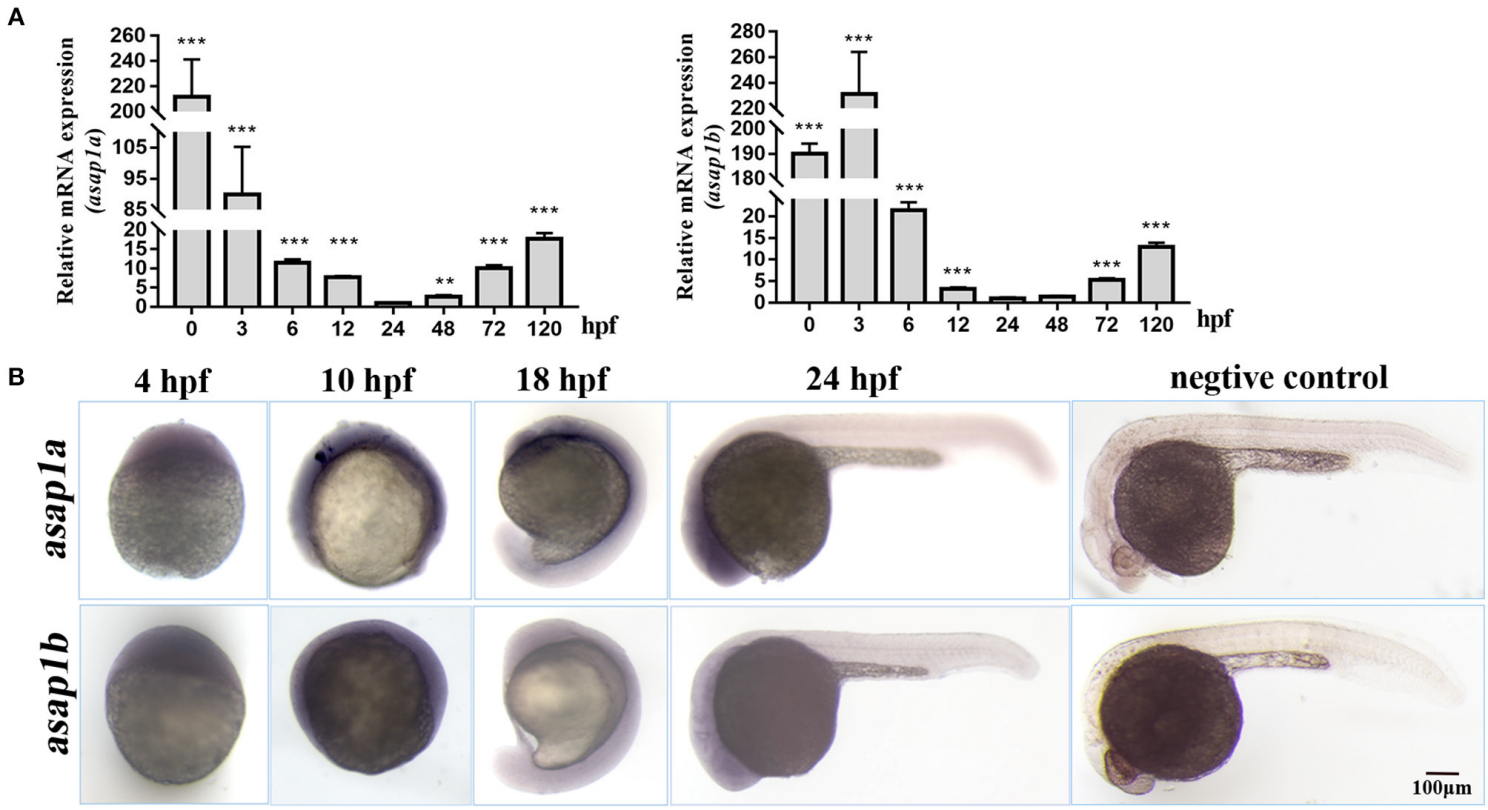
Expression pattern of zebrafish *asap1* homologs during embryonic development. **(A)** Temporal expression profiles of *asap1a* and *asap1b* by qRT-PCR. **P < 0.01, ***P < 0.001 vs. 24 hpf control group using Student's unpaired *t*-test. Data shown are representative of 3 independent experiments. **(B)** Spatial expression of *asap1a* (upper panels) and *asap1b* (lower panels) by whole mount *in situ* hybridization in wild-type embryos, the sense probe served as negative control (Scale bar, 100 μm).

To identify the zebrafish *asap1* genes, the human ASAP1 protein sequence was used as the reference to perform the p-n Blast against zebrafish genomes (GRCz11). In contrast to the single human *ASAP1* gene, we found two hits (*asap1a* and *asap1b*) in the zebrafish genome. Localized on chromosome 2, *asap1a* contains 29 exons and encodes a protein of 1151 amino acids, while *asap1b* on chromosome 24 contains 29 exons and encodes a protein of 1140 amino acids. Primary structure analysis shows that both zebrafish Asap1a and Asap1b share identical functional domains with human ASAP1, including BAR (Bin, Amphiphysin and Rvs167 and Rvs161), ArfGAP (ADP ribosylation factor-GTPase activating protein), SH3 (Src Homolog 3), ANK (ankyrin) repeat, and PH (Pleckstrin Homology) domains. Zebrafish Asap1a and Asap1b share ~76 and 78% identity with human ASAP1, respectively (Figure 1). Syntenic and phylogenetic analysis reveals that zebrafish *asap1a* and *asap1b* are evolutionarily conserved in their chromosome linkage positions and evolutionary relationship (Supplementary Figure 1). Through temporal expression lapse, we found *asap1a* and *asap1b* mRNA major concentrated at 3 hpf, suggesting that *asap1* may be under maternal control (Figure 2A). However, the mRNA of *asap1a* and *asap1b* were at a low level from 12 to 48 hpf, and subsequently increased from 72 hpf according to the qRT-PCR analysis. Whole mount *in situ* hybridization demonstrates that *asap1a and asap1b* transcripts are ubiquitously distributed at early stages of 4 and 10 hpf, but the expression of *asap1a and asap1b* are getting weaker at 18 and 24 hpf, especially in the tail muscle tissue at 24 hpf (Figure 2B).

### *asap1* Morphants Are Susceptible to *Mm* Infection

**Figure 3:**
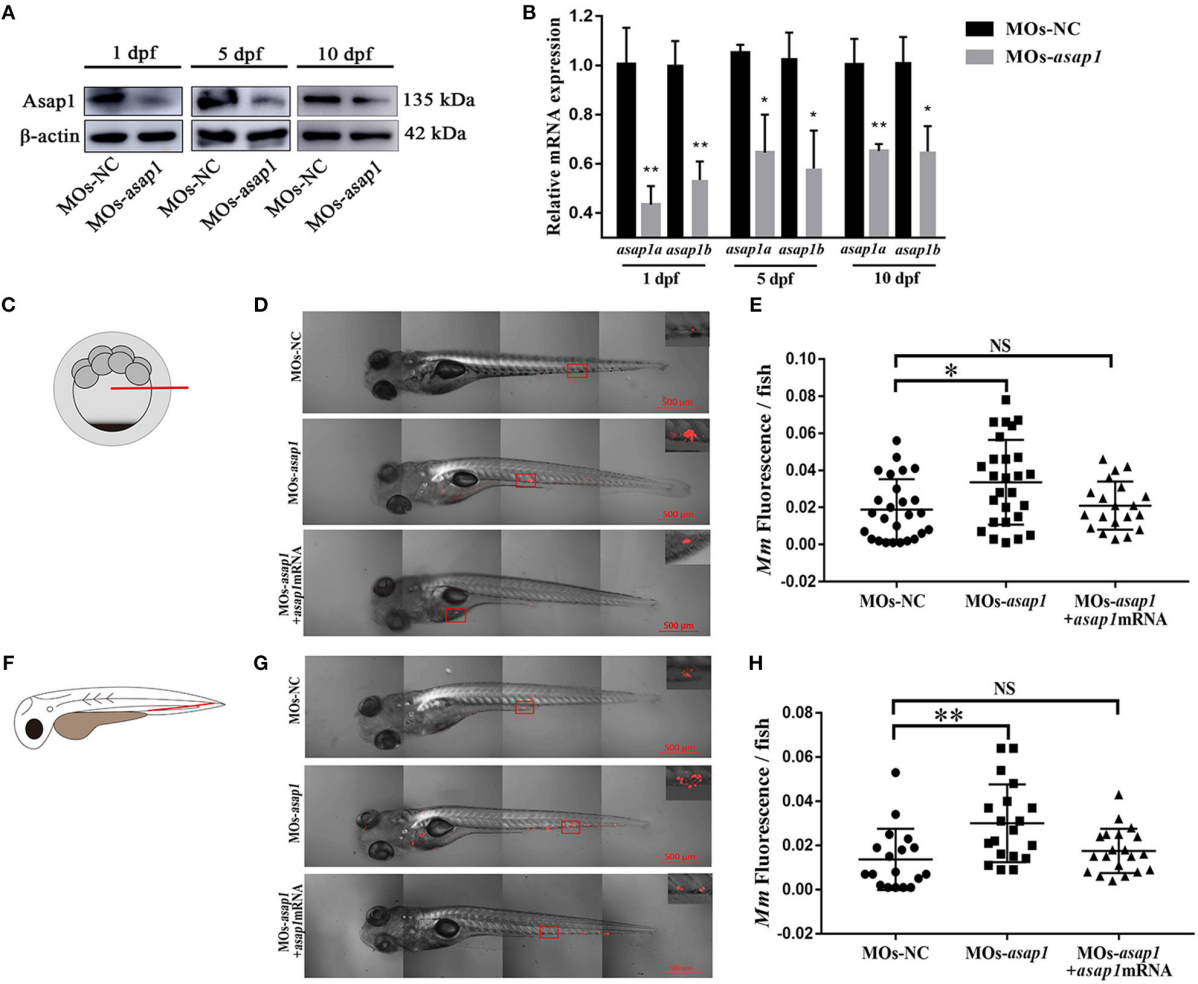
*asap1* morphants exhibit altered susceptibility to *Mm* infection. **(A,B)** Western blotting **(A)** and qRT-PCR **(B)** analysis of the knockdown efficiency of *asap1* morphants in 1, 5, and 10 dpf zebrafish. *P < 0.05, **P < 0.01 vs. control morphants using Student's unpaired *t*-test. The antibody was against both Asap1a and Asap1b. Data shown are representative of 3 independent experiments. **(C)** Schematic diagram of the yolk injection method. **(D,E)** Representative fluorescence images and quantification of bacterial burdens by fluorescence pixel counts of controls, *asap1* morphants, or *asap1* morphants treated with *asap1* mRNA at 5 dpi with equivalent bacterial inocula by the yolk injection method (scale bar 500 μm); *P < 0.05, *P*-values calculated by Kruskal-Wallis with Dunn's post-test. *n* (MOs-NC group) = 27, *n* (MOs-*asap1* group) = 27, *n* (MOs-*asap1*+*asap1*mRNA group) = 20. Mean ± SEM from four pooled independent experiments. **(F)** Schematic diagram of the intravenous injection method. **(G,H)** Representative fluorescence images and quantification of bacterial burdens by fluorescence pixel counts of controls, *asap1* morphants or *asap1* morphants treated with *asap1* mRNA at 5 dpi with equivalent bacterial inocula by the intravenous injection method (scale bar, 500 μm); **P < 0.01, NS, not significant, *P*-values calculated by Kruskal-Wallis with Dunn's post-test. *n* (MOs-NC group) = 18, *n* (MOs-*asap1* group) = 19, *n* (MOs-*asap1*+*asap1*mRNA group) = 20. Each dot represents one larva. Mean ± SEM from three pooled independent experiments. MOs, morpholino oligonucleotide mixture. *asap1* means *asap1a* and *asap1b*.

To explore the function of Asap1 in zebrafish against *Mm* infection, firstly the function model of zebrafish *asap1* loss was generated by morpholino mediated knockdown methods (Stainier et al., 2017). By injecting of specific anti-sense morpholinos targeting the exon 1 of premature *asap1a* and *asap1b* mRNA, we successfully detected high efficiency knockdown for *asap1* until 10 dpf by qRT-PCR and western blotting (Figures 3A,B), which makes the *asap1* morphants acceptable for follow-up studies. Notably, the Asap1 antibody used in this experiment was derived from a recombinant peptide of 20 amino acids corresponding to the mouse ASAP1 protein, and the antigen region was highly conserved in zebrafish Asap1 proteins (Figure 1). Thus, it was supposed to recognize both Asap1a and Asap1b. The result of western blotting revealed that we successfully detected the expression level of the zebrafish protein but failed to distinguish the Asap1 homologs, owing to the high similarity of the two Asap1 proteins. However, all morphants developed normally and didn't show any obvious morphological defects within 2 weeks. Next, to evaluate the roles of *asap1* against *Mm* infection *in vivo, Mm* were firstly injected into the yolk of morphant embryos at the 16–1,000 cells stage (Figure 3C). We then followed existing protocol to detect tdTomato-*Mm* fluorescent expression in 5 dpi zebrafish (Benard et al., 2012; Takaki et al., 2013). Preliminary experimental results showed that Asap1 knockdown enhanced *Mm* infection efficiency by ~2-fold (Figures 3D,E), but a single knockdown of Asap1a or Asap1b did not cause increased susceptibility to *Mm* over controls (Supplementary Figures 2A–D). Intravenous injection with *Mm* is commonly used to evaluate the infection in zebrafish embryos (Figure 3F). Here, *Mm* was also intravenously injected into 28–32 hpf embryos after treatment with control or *asap1* MOs. The results also showed that compared with the control group, *asap1* morphants markedly increased the infection efficiency of *Mm* with 2.5-fold more bacteria (Figures 3G,H) but *asap1a* or *asap1b* morphants did not (Supplementary Figures 2E–H). Meanwhile, we also found that mortality rate did not significantly differ (no statistical difference) among *Mm* infected morphants no matter whether it was introduced through yolk (Supplementary Figure 3A) or intravenous injection (Supplementary Figure 3B). Additionally, to definitively demonstrate that the loss of Asap1 was causative for susceptibility phenotype, we performed a rescue experiment in *asap1* morphants injecting with synthetic *asap1a* and *asap1b* mRNA. Expectedly, the re-expression of *asap1a* and *asap1b* in zebrafish, as evidenced by the qRT-PCR and western blotting (Supplementary Figure 4), can partially rescue the susceptibility of *asap1* morphants to *Mm* infection and the survival of zebrafish post-infection (Figures 3D–H, Supplementary Figure 3A,B). Taken together, these results showed that knockdown of Asap1 as a whole weakened zebrafish resistance to *Mm* infection even if they didn't obviously affect the survival status within 10 d post-infection.

### *asap1* Morphants Exhibit Delayed Macrophage Recruitment

**Figure 4:**
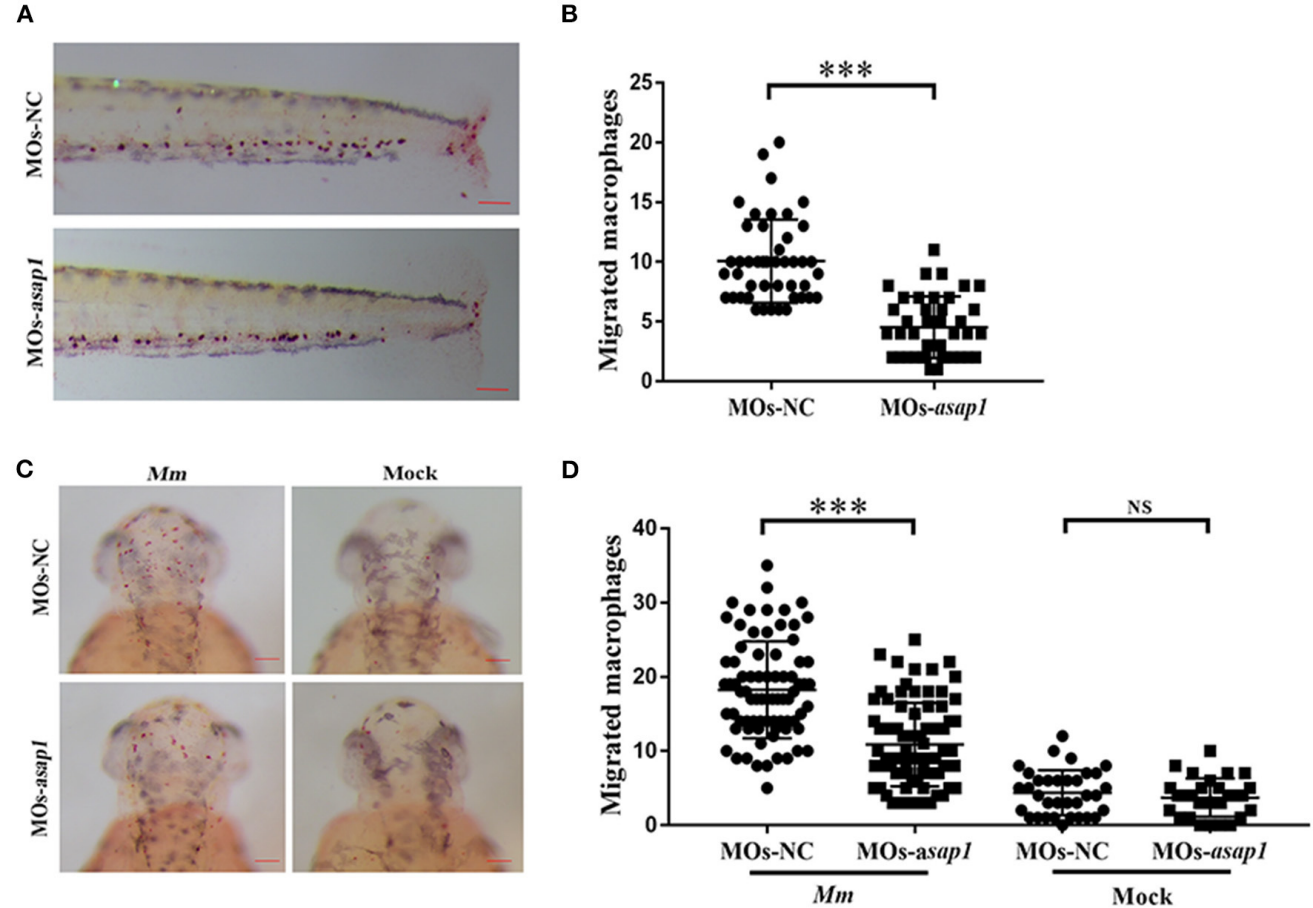
*asap1* morphant macrophages exhibit impaired migration. **(A)** Representative light microscopy images of neutral red stained macrophages of transgenic morphants migrated to tailfin injury at 9 h post injury. **(B)** Numbers of recruited neutral red stained macrophages in the tailfin injury at 9 h post injury (scale bar, 100 μm); *** *P* < 0.001, *P*-values calculated by Student's unpaired *t*-test. *n* (MOs-NC group) = 45, *n* (MOs-*asap1* group) = 40. **(C)** Representative light microscopy images of neutral red stained macrophages following hindbrain injection with *Mm* (left panels) or mock (right panels) into morphants at 9 h post infection. **(D)** Numbers of recruited neutral red stained macrophages in the hindbrain at 9 h post infection (scale bar, 100 μm); ***P < 0.001, NS, not significant, *P*-values calculated between two groups by Student's unpaired *t*-test. *n* (MOs-NC group/*Mm*) = 77, *n* (MOs-*asap1* group/*Mm*) = 81, *n* (MOs-NC group/mock) =34, *n* (MOs-*asap1* group/mock) =29. Each dot represents one larva. Mean ± SEM from two pooled independent experiments. MOs, morpholino oligonucleotide mixture. *asap1* means *asap1a* and *asap1b*.

A previous study showed that ASAP1 was involved in regulating dendritic cell migration (Curtis et al., 2015). In the zebrafish infectious model, neutrophils and macrophages are the primary cells to act against pathogens (Bernut et al., 2016; Rosowski et al., 2016). To dissect the mechanism of Asap1 in regulating zebrafish against infection, we further examined the effect of Asap1 on macrophage migration in zebrafish by tailfin click and *Mm* hindbrain injection. In tailfin transection assay, *asap1* morphants exhibited significant defects of macrophage migration in response to injury (Figures 4A,B). The hindbrain ventricle is a suitable site to evaluate macrophage recruitment toward local infections (Benard et al., 2012; Pagan et al., 2015). When we further injected hindbrain ventricles of *asap1* morphants with *Mm*, we noted that far fewer macrophages were recruited and crossed the brain-blood barrier than in control morphants (Figures 4C,D). Meanwhile, we confirmed that total macrophage numbers did not differ between *asap1* and control morphants (Supplementary Figure 5), using a published method that quantified neutral red stained macrophages in whole zebrafish larvae (Tobin et al., 2010). These results suggest that impaired macrophage migration is not due to a reduction in total macrophage number.

### Knockdown of ASAP1 Impaired THP-1 Cells Migration

**Figure 5:**
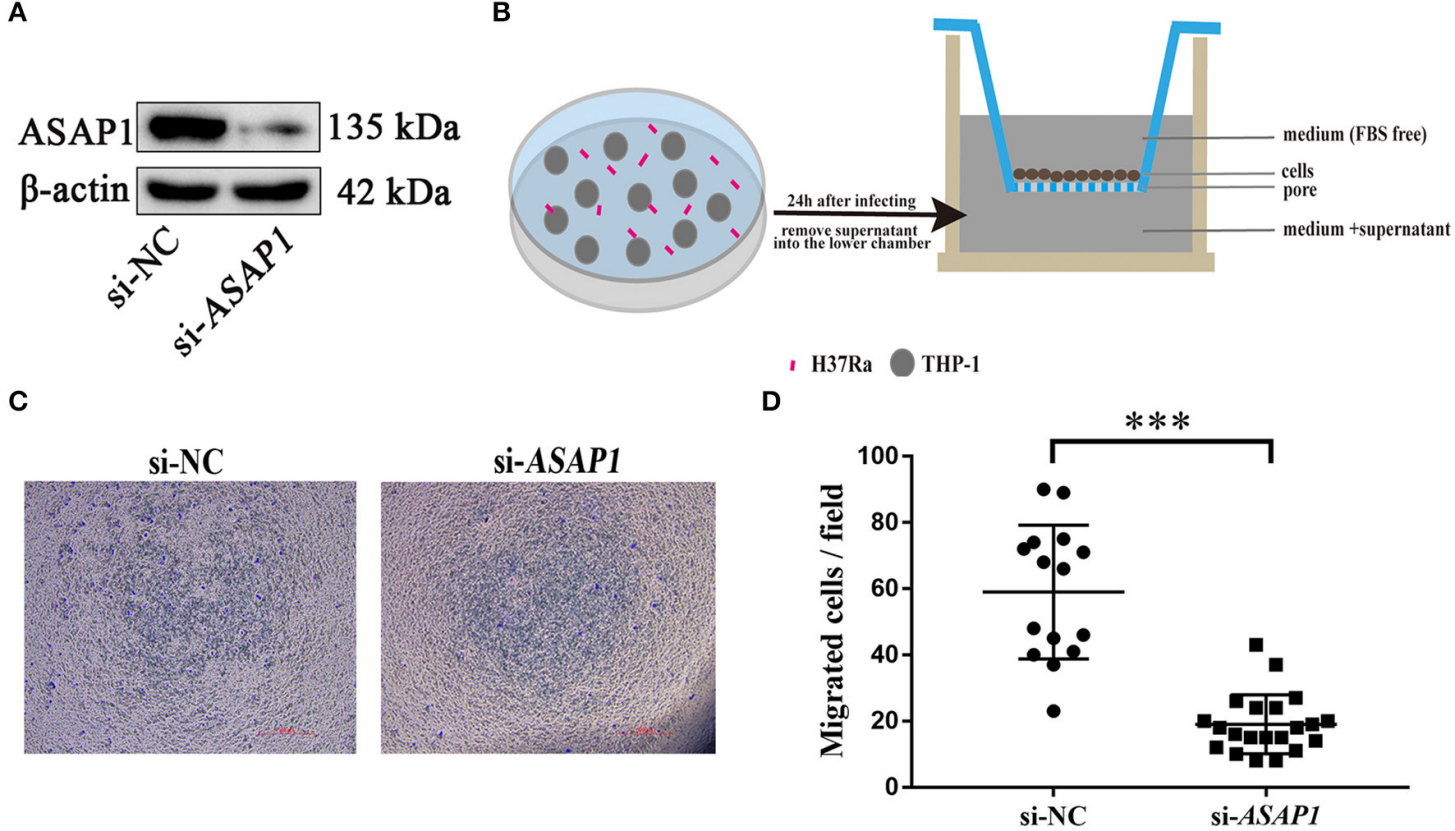
Migration of cells is impaired in THP-1 cells upon knockdown of ASAP1. **(A)** Western blotting analysis of the knockdown efficiency of ASAP1 when THP-1 cells were transfected with control or *ASAP1* siRNA. **(B)** Schematic diagram of the Transwell protocol. **(C,D)** Transwell assay was applied to evaluate the effect of ASAP1 on cell migration (scale bar, 500 μm); ***P < 0.001, *P*-values calculated by the Mann-Whitney test. Each dot represents one scope. Mean ± SEM from two independent experiments.

To examine the role of ASAP1 in regulating macrophage motility, THP-1 cells were transfected with either siRNA against *ASAP1* or control siRNA, and then subjected to the Transwell assay to determine cell migration ability. Western blotting analysis revealed that ASAP1 was knocked down successfully (Figure 5A). At 24 h post treatment with *ASAP1* siRNA or control siRNA, the cells were transferred to the upper chamber of a Transwell culture plate. Then, the medium supernatant of THP-1 cells infected with H37Ra for 24 h was added to the lower chamber (Figure 5B). The results showed that ASAP1 knockdown THP-1 cells exhibited reduced migration ability compared to that of their control-transfected counterparts (Figures 5C,D).

## Discussion

In our work, we initially characterized zebrafish *asap1a* and *asap1b*, both of encoding proteins with very high amino-acid sequence identity to mammalian ASAP1. We then found that Asap1 deficiency in zebrafish significantly enhanced susceptibility to *Mm*, which could be rescued by injecting with *asap1* mRNA. To fully validate susceptibility phenotypes, we compared two different mycobacterial injection methods for evaluating the Asap1-mediated susceptibility to TB: yolk injection and intravenous injection. In the former method, *Mm* were injected into 16–1,000 cell embryos, which affords easy manipulation but confers a high mortality rate. In the latter method, *Mm* were intravenously injected into 28–32 h embryos, which represents the optimal method for researching *Mm* susceptibility in zebrafish owing to the formation of a rapid systemic infection and allowing precise fluorescence-based measurements of overall bacterial burden. Additionally, these two different zebrafish *asap1* isoforms, *asap1a* and *asap1b*, did not alter the susceptibility of zebrafish to *Mm* when they were knocked down separately, indicating that both of these proteins contribute to susceptibility phenotypes and may have partially overlapping functions in zebrafish. Furthermore, *asap1a* and *asap1b* share 76% identity in protein sequence with each other (Figure 1), which makes it difficult to distinguish the different functions and the redundant degree between *asap1a* and *asap1b*. Therefore, it is still unclear whether the roles of *asap1a* and *asap1b* are overlapping or distinct. Here, we should point out that mortality rate in *asap1* morphants was not significantly higher than that in the controls after an increase in bacterial load, unlike *lta4h* morphants and *csf1r* mutants (Tobin et al., 2010; Pagan et al., 2015). This outcome suggests that Asap1-induced elevation in bacterial load might not strongly influence infection lethality initiated via normal *Mm* inocula, at least in the zebrafish model. The lack of an effect on mortality rates may also be due to differences in the complex progressive disease of TB across hosts. Future studies should explore in greater detail for the association between bacterial load and TB pathological mechanisms when Asap1 is deleted in zebrafish. Despite the need for further elucidation of mechanisms and infection processes, our zebrafish model highlights the importance of ASAP1 in host resistance to mycobacterial entry.

*ASAP1* polymorphisms are associated with TB susceptibility in many populations (Curtis et al., 2015; Wang et al., 2018; Chen et al., 2019). But we know little about how *ASAP1* polymorphisms confer altered TB susceptibility or the underlying pathophysiological mechanisms. Moreover, currently recognized *ASAP1* polymorphisms associated with TB susceptibility are all intronic, increasing the difficulty of analyzing the relationship between ASAP1 and TB susceptibility. Genetic defects in ASAP1 mediate susceptibility to *Salmonella* infections, but a similar connection has not been investigated for *Mycobacterium*. The ArfGEF (Arf Guanine-nucleotide exchange factor) and ArfGAP cycles ensure the alternation of Arf activation and deactivation, which jointly regulate pathogens entry into cells (Humphreys et al., 2013). Recent findings have illustrated that ArfGAP engages to modulate mycobacterial colonization by controlling the actin cytoskeleton (Song et al., 2018). It appears possible that bacteria might interfere with ArfGAP in order to sustain activation of Arf, which is required for rearrangements of the actin cytoskeleton and facilitates bacterial invasion via the cell membrane surface complex, such as through ruffles and podosomes (Bharti et al., 2007; Rafiq et al., 2017). Therefore, we examined the role of ASAP1 on the migration effect of phagocytes to *Mm* infection in the zebrafish model.

Macrophages phagocytose *Mtb* and then secrete a large number of inflammatory factors to recruit other macrophages or immune cells to form an organized granuloma structure, which constitutes the initial reaction in TB disease and benefits the host through the control of *Mtb* in the body and the limitations of the lesion. However, from another perspective, once macrophages parasitized by *Mtb* in the granuloma become necrotic, bacteria are released, and spread to the whole body, such that the macrophages and granuloma actually function as a bacterial multiplicative tool for expanding infection (Cambier et al., 2014; Guirado et al., 2015; Martin et al., 2016). Nevertheless, the ability of macrophages in immune response to kill *Mtb* is crucial in terms of fighting TB. A 2016 study reported that inhibition of macrophage migration in zebrafish delayed phagocytosis of *Mm*, thus potentially driving susceptibility to TB (Berg et al., 2016). In the present study, we found that the knockdown of Asap1 in zebrafish significantly decreased the migration of macrophages during *Mm* infection, moreover, similar result was obtained using THP-1 cells as determined with a Transwell assay, which together strongly indicated that ASAP1 regulated the migration of macrophages. We further suppose that such impaired migration of macrophages may result in a slow microbicidal response or increase the time required to initiate an effectively adaptive immune response for *Mycobacterium*, thereby inducing a weak innate and adaptive immune response to control mycobacterial proliferation in zebrafish larvae. In contrast, Torraca et al. (2015) and Sommer et al. (2020) reported that diminished macrophage migration caused by *cxcr3* depletion might limit dissemination of mycobacterial infection and lead to an reduction in total bacterial burden in zebrafish. Basing on the above viewpoints, it will therefore be of great interest to investigate how macrophages interact with *Mm* in the context of ASAP1 deletion, using adult zebrafish with mature immune system. Together, Our work highlights the importance of ASAP1 in modulating the balance between innate immune control and mycobacterial infection and suggests a mechanistic explanation for the reported GWAS findings between ASAP1 and TB susceptibility. Moreover, our findings may provide an explanation regarding how a single genetic determinant might influence antimicrobial and immunological dysfunction of phagocytic cells. Considering a further finding that ArfGEFs and ArfGAPs might collaborate to control pathogen infection (Liu et al., 2005; Davidson et al., 2015), we speculate that ASAP1-mediated regulation of mycobacterial invasion occurs through various temporally and spatially adjusted mechanisms. Although herein we reveal only the basic morphology and give a preliminary description of the relationship between ASAP1 and *Mycobacterium*, the results of zebrafish Asap1 provide a new perspective on the debate regarding host susceptibility genes and *Mycobacterium* infection, which may facilitate the development of improved preventive and therapeutic strategies for TB. Toward this end, further detailed examination of the mechanisms underlying ASAP1 function in regulating mycobacterial invasion and persistence in macrophages is warranted.

## Data Availability Statement

All datasets generated for this study are included in the article/Supplementary Material.

## Ethics Statement

The Ethics Committee of Shanxi University approved this study (SXULL2019004). Written informed consent was obtained from the owners for the participation of their animals in this study.

## Author Contributions

ZZ and CW contributed to project conception and supervised the projects. JC, DW, and YW performed experiments. JC, GC, and ZZ performed data analysis and statistical tests and wrote the manuscript. All authors contributed to manuscript revision and approved the final submitted manuscript.

## Conflict of Interest

The authors declare that the research was conducted in the absence of any commercial or financial relationships that could be construed as a potential conflict of interest.

## References

[B1] Behar S. M. Martin C. J. Booty M. G. Nishimura T. Zhao X. Gan H. X.. (2011). Apoptosis is an innate defense function of macrophages against *Mycobacterium tuberculosis*. Mucosal. Immunol. 4, 279–287. 10.1038/mi.2011.3 21307848 PMC3155700

[B2] Benard E. L. Van Der Sar A. M. Ellett F. Lieschke G. J. Spaink H. P. Meijer A. H. (2012). Infection of zebrafish embryos with intracellular bacterial pathogens. J. Vis. Exp. 61:3781. 10.3791/3781 22453760 PMC3415172

[B3] Berg R. D. Levitte S. O'sullivan M. P. O'leary S. M. Cambier C. J. Cameron J.. (2016). Lysosomal disorders drive susceptibility to tuberculosis by compromising macrophage migration. Cell 165, 139–152. 10.1016/j.cell.2016.02.034 27015311 PMC4819607

[B4] Bernut A. Nguyen-Chi M. Halloum I. Herrmann J. L. Lutfalla G. Kremer L. (2016). *Mycobacterium abscessus*-induced granuloma formation is strictly dependent on TNF signaling and neutrophil trafficking. PLoS Pathog. 12:e1005986. 10.1371/journal.ppat.1005986 27806130 PMC5091842

[B5] Bharti S. Inoue H. Bharti K. Hirsch D. S. Nie Z. Yoon H. Y.. (2007). Src-dependent phosphorylation of ASAP1 regulates podosomes. Mol. Cell. Biol. 27, 8271–8283. 10.1128/MCB.01781-06 17893324 PMC2169185

[B6] Cambier C. J. Takaki K. K. Larson R. P. Hernandez R. E. Tobin D. M. Urdahl K. B.. (2014). Mycobacteria manipulate macrophage recruitment through coordinated use of membrane lipids. Nature 505, 218–222. 10.1038/nature12799 24336213 PMC3961847

[B7] Chen C. Zhao Q. Shao Y. Li Y. Song H. Li G.. (2019). A Common variant of ASAP1 is associated with tuberculosis susceptibility in the han chinese population. Dis. Markers 2019:7945429. 10.1155/2019/7945429 31089398 PMC6476032

[B8] Clay H. Volkman H. E. Ramakrishnan L. (2008). Tumor necrosis factor signaling mediates resistance to Mycobacteria by inhibiting bacterial growth and macrophage death. Immunity 29, 283–294. 10.1016/j.immuni.2008.06.011 18691913 PMC3136176

[B9] Cronan M. R. Tobin D. M. (2014). Fit for consumption: zebrafish as a model for tuberculosis. Dis. Model Mech. 7, 777–784. 10.1242/dmm.016089 24973748 PMC4073268

[B10] Curtis J. Luo Y. Zenner H. L. Cuchet-Lourenco D. Wu C. Lo K.. (2015). Susceptibility to tuberculosis is associated with variants in the ASAP1 gene encoding a regulator of dendritic cell migration. Nat. Genet. 47, 523–527. 10.1038/ng.3248 25774636 PMC4414475

[B11] Das R. Koo M. S. Kim B. H. Jacob S. T. Subbian S. Yao J.. (2013). Macrophage migration inhibitory factor (MIF) is a critical mediator of the innate immune response to *Mycobacterium tuberculosis*. Proc. Natl. Acad. Sci. U.S.A. 110, E2997–3006. 10.1073/pnas.1301128110 23882081 PMC3740876

[B12] Davidson A. C. Humphreys D. Brooks A. B. Hume P. J. Koronakis V. (2015). The arf GTPase-activating protein family is exploited by Salmonella enterica serovar Typhimurium to invade nonphagocytic host cells. mBio. 6:14. 10.1128/mBio.02253-14 25670778 PMC4337568

[B13] Davis J. M. Ramakrishnan L. (2009). The role of the granuloma in expansion and dissemination of early tuberculous infection. Cell 136, 37–49. 10.1016/j.cell.2008.11.014 19135887 PMC3134310

[B14] Guirado E. Mbawuike U. Keiser T. L. Arcos J. Azad A. K. Wang S. H.. (2015). Characterization of host and microbial determinants in individuals with latent tuberculosis infection using a human granuloma model. MBio 6, e02537–e02514. 10.1128/mBio.02537-14 25691598 PMC4337582

[B15] Gutzman J. H. Sive H. (2009). Zebrafish brain ventricle injection. J. Vis. Exp. 26:1218. 10.3791/1218 19352312 PMC2791086

[B16] Helguera-Repetto C. Cox R. A. Munoz-Sanchez J. L. Gonzalez-Y-Merchand J. A. (2004). The pathogen Mycobacterium marinum, a faster growing close relative of *Mycobacterium tuberculosis*, has a single rRNA operon per genome. FEMS Microbiol. Lett. 235, 281–288. 10.1111/j.1574-6968.2004.tb09600.x 15183875

[B17] Herbomel P. Thisse B. Thisse C. (2001). Zebrafish early macrophages colonize cephalic mesenchyme and developing brain, retina, and epidermis through a M-CSF receptor-dependent invasive process. Dev. Biol. 238, 274–288. 10.1006/dbio.2001.0393 11784010

[B18] Huang Z. Su R. Deng Z. Xu J. Peng Y. Luo Q.. (2017). Identification of differentially expressed circular RNAs in human monocyte derived macrophages response to *Mycobacterium tuberculosis* infection. Sci. Rep. 7:13673. 10.1038/s41598-017-13885-0 29057952 PMC5651861

[B19] Humphreys D. Davidson A. C. Hume P. J. Makin L. E. Koronakis V. (2013). Arf6 coordinates actin assembly through the WAVE complex, a mechanism usurped by Salmonella to invade host cells. Proc. Natl. Acad. Sci. U.S.A. 110, 16880–16885. 10.1073/pnas.1311680110 24085844 PMC3801044

[B20] Korb V. C. Chuturgoon A. A. Moodley D. (2016). *Mycobacterium tuberculosis*: manipulator of protective immunity. Int. J. Mol. Sci. 17:131. 10.3390/ijms17030131 26927066 PMC4813124

[B21] Lewinsohn D. M. Lewinsohn D. A. (2019). New concepts in tuberculosis host defense. Clin. Chest. Med. 40, 703–719. 10.1016/j.ccm.2019.07.002 31731979

[B22] Liu Y. Loijens J. C. Martin K. H. Karginov A. V. Parsons J. T. (2002). The association of ASAP1, an ADP ribosylation factor-GTPase activating protein, with focal adhesion kinase contributes to the process of focal adhesion assembly. Mol. Biol. Cell. 13, 2147–2156. 10.1091/mbc.e02-01-0018 12058076 PMC117631

[B23] Liu Y. Yerushalmi G. M. Grigera P. R. Parsons J. T. (2005). Mislocalization or reduced expression of Arf GTPase-activating protein ASAP1 inhibits cell spreading and migration by influencing Arf1 GTPase cycling. J. Biol. Chem. 280, 8884–8892. 10.1074/jbc.M412200200 15632162

[B24] Marino S. Pawar S. Fuller C. L. Reinhart T. A. Flynn J. L. Kirschner D. E. (2004). Dendritic cell trafficking and antigen presentation in the human immune response to *Mycobacterium tuberculosis*. J. Immunol. 173, 494–506. 10.4049/jimmunol.173.1.494 15210810

[B25] Martin C. J. Carey A. F. Fortune S. M. (2016). A bug's life in the granuloma. Semin. Immunopathol. 38, 213–220. 10.1007/s00281-015-0533-1 26577238 PMC4834868

[B26] Moule M. G. Cirillo J. D. (2020). Mycobacterium Tuberculosis dissemination plays a critical role in pathogenesis. Front. Cell Infect. Microbiol. 10:65. 10.3389/fcimb.2020.00065 32161724 PMC7053427

[B27] Nathan C. (2016). Macrophages' choice: take it in or keep it out. Immunity. 45, 710–711. 10.1016/j.immuni.2016.10.002 27760333

[B28] Nguyen-Chi M. Laplace-Builhe B. Travnickova J. Luz-Crawford P. Tejedor G. Phan Q. T.. (2015). Identification of polarized macrophage subsets in zebrafish. Elife. 4:e07288. 10.7554/eLife.07288 26154973 PMC4521581

[B29] Pagan A. J. Yang C. T. Cameron J. Swaim L. E. Ellett F. Lieschke G. J.. (2015). Myeloid growth factors promote resistance to mycobacterial infection by curtailing granuloma necrosis through macrophage replenishment. Cell Host Microbe. 18, 15–26. 10.1016/j.chom.2015.06.008 26159717 PMC4509513

[B30] Rafiq N. B. Lieu Z. Z. Jiang T. Yu C. H. Matsudaira P. Jones G. E.. (2017). Podosome assembly is controlled by the GTPase ARF1 and its nucleotide exchange factor ARNO. J. Cell. Biol. 216, 181–197. 10.1083/jcb.201605104 28007915 PMC5223603

[B31] Randazzo P. A. Andrade J. Miura K. Brown M. T. Long Y. Q. Stauffer S.. (2000). The Arf GTPase-activating protein ASAP1 regulates the actin cytoskeleton. Proc. Nat. Acad. Sci. U.S.A. 97, 4011–4016. 10.1073/pnas.070552297 10725410 PMC18133

[B32] Randazzo P. A. Inoue H. Bharti S. (2007). Arf GAPs as regulators of the actin cytoskeleton. Biol. Cell. 99, 583–600. 10.1042/BC20070034 17868031

[B33] Roca F. J. Ramakrishnan L. (2013). TNF dually mediates resistance and susceptibility to mycobacteria via mitochondrial reactive oxygen species. Cell. 153, 521–534. 10.1016/j.cell.2013.03.022 23582643 PMC3790588

[B34] Rosen J. N. Sweeney M. F. Mably J. D. (2009). Microinjection of zebrafish embryos to analyze gene function. J. Vis. Exp. 2009:1115. 10.3791/1115 19274045 PMC2762901

[B35] Rosowski E. E. Deng Q. Keller N. P. Huttenlocher A. (2016). Rac2 functions in both neutrophils and macrophages to mediate motility and host defense in larval zebrafish. J. Immunol. 197, 4780–4790. 10.4049/jimmunol.1600928 27837107 PMC5367389

[B36] Sanderson L. E. Chien A. T. Astin J. W. Crosier K. E. Crosier P. S. Hall C. J. (2015). An inducible transgene reports activation of macrophages in live zebrafish larvae. Dev. Comp. Immunol. 53, 63–69. 10.1016/j.dci.2015.06.013 26123890

[B37] Schreiber C. Saraswati S. Harkins S. Gruber A. Cremers N. Thiele W.. (2019). Loss of ASAP1 in mice impairs adipogenic and osteogenic differentiation of mesenchymal progenitor cells through dysregulation of FAK/Src and AKT signaling. PLoS Genet. 15:e1008216. 10.1371/journal.pgen.1008216 31246957 PMC6619832

[B38] Shiau C. E. Kaufman Z. Meireles A. M. Talbot W. S. (2015). Differential requirement for irf8 in formation of embryonic and adult macrophages in zebrafish. PLoS ONE 10:e0117513. 10.1371/journal.pone.0117513 25615614 PMC4304715

[B39] Silva Miranda M. Breiman A. Allain S. Deknuydt F. Altare F. (2012). The tuberculous granuloma: an unsuccessful host defence mechanism providing a safety shelter for the bacteria? Clin. Dev. Immunol. 2012:139127. 10.1155/2012/139127 22811737 PMC3395138

[B40] Smith M. P. Young H. Hurlstone A. Wellbrock C. (2015). Differentiation of THP1 cells into macrophages for transwell co-culture assay with melanoma cells. Bio. Protoc. 5:1638. 10.21769/BioProtoc.1638 27034969 PMC4811304

[B41] Sommer F. Torraca V. Kamel S. M. Lombardi A. Meijer A. H. (2020). Frontline science: antagonism between regular and atypical Cxcr3 receptors regulates macrophage migration during infection and injury in zebrafish. J. Leukoc. Biol. 107, 185–203. 10.1002/JLB.2HI0119-006R 31529512 PMC7028096

[B42] Song O. R. Queval C. J. Iantomasi R. Delorme V. Marion S. Veyron-Churlet R.. (2018). ArfGAP1 restricts *Mycobacterium tuberculosis* entry by controlling the actin cytoskeleton. EMBO Rep. 19, 29–42. 10.15252/embr.201744371 29141986 PMC5757213

[B43] Stainier D. Y. R. Raz E. Lawson N. D. Ekker S. C. Burdine R. D. Eisen J. S.. (2017). Guidelines for morpholino use in zebrafish. PLoS Genet. 13:e1007000. 10.1371/journal.pgen.1007000 29049395 PMC5648102

[B44] Takaki K. Davis J. M. Winglee K. Ramakrishnan L. (2013). Evaluation of the pathogenesis and treatment of mycobacterium marinum infection in zebrafish. Nat. Protoc. 8, 1114–1124. 10.1038/nprot.2013.068 23680983 PMC3919459

[B45] Tien D. N. Kishihata M. Yoshikawa A. Hashimoto A. Sabe H. Nishi E.. (2014). AMAP1 as a negative-feedback regulator of nuclear factor-κB under inflammatory conditions. Sci. Rep. 4:5094. 10.1038/srep05094 24865276 PMC4035583

[B46] Tobin D. M. Ramakrishnan L. (2008). Comparative pathogenesis of mycobacterium marinum and *Mycobacterium tuberculosis*. Cell Microbiol. 10, 1027–1039. 10.1111/j.1462-5822.2008.01133.x 18298637

[B47] Tobin D. M. Vary J. C. Jr. Ray J. P. Walsh G. S. Dunstan S. J. Bang N. D.. (2010). The lta4h locus modulates susceptibility to mycobacterial infection in zebrafish and humans. Cell. 140, 717–730. 10.1016/j.cell.2010.02.013 20211140 PMC2907082

[B48] Torraca V. Cui C. Boland R. Bebelman J. P. Van Der Sar A. M. Smit M. J.. (2015). The CXCR3-CXCL11 signaling axis mediates macrophage recruitment and dissemination of mycobacterial infection. Dis. Model Mech. 8, 253–269. 10.1242/dmm.017756 25573892 PMC4348563

[B49] van der Vaart M. Van Soest J. J. Spaink H. P. Meijer A. H. (2013). Functional analysis of a zebrafish myd88 mutant identifies key transcriptional components of the innate immune system. Dis. Model Mech. 6, 841–854. 10.1242/dmm.010843 23471913 PMC3634667

[B50] Wang X. Ma A. Han X. Litifu A. Xue F. (2018). ASAP1 gene polymorphisms are associated with susceptibility to tuberculosis in a Chinese Xinjiang Muslim population. Exp. Ther. Med. 15, 3392–3398. 10.3892/etm.2018.5800 29545860 PMC5841074

[B51] WHO (2019). Global Tuberculosis Report 2019. Geneva: World Health Organization. Available online at: https://www.who.int/tb/en/

[B52] Yuan S. Sun Z. (2009). Microinjection of mRNA and morpholino antisense oligonucleotides in zebrafish embryos. J. Vis. Exp. 2009:1113. 10.3791/1113 19488022 PMC2762915

