# Supplementary material for: Asap1 Affects the Susceptibility of Zebrafish to Mycobacterium by Regulating Macrophage Migration
Source: Front Cell Infect Microbiol. 2020 Oct 29;10:519503. doi: 10.3389/fcimb.2020.519503 (PMC7658321; doi:10.3389/fcimb.2020.519503)
Supplement: Supplementary file 1 [file Data_Sheet_1.docx]

Supplementary Material

# Supplementary Figures

**Supplementary Figure 1 │** [Bioinformatics](https://cn.bing.com/dict/search?q=Bioinformatics&FORM=BDVSP6&mkt=zh-cn) [analysis](https://cn.bing.com/dict/search?q=Analysis&FORM=BDVSP6&mkt=zh-cn) [of](https://cn.bing.com/dict/search?q=of&FORM=BDVSP6&mkt=zh-cn) the *ASAP1* gene and ASAP1 homologs. (**A**) Schematic diagram of syntenic analysis of human *ASAP1,* mouse *Asap1* and zebrafish *asap1a* and *asap1b*. (**B**) A phylogenetic tree was constructed with the neighbor-joining algorithm using the Mega X program ([www.megasoftware.net](http://www.megasoftware.net)) based on the multiple sequence alignments. The percentage of replication trees where the associated taxa are clustered together in 500 bootstraps are shown next to the branches. *Homo sapiens* ASAP1 (UniProt: A0A0A0MRE5), *Pan troglodytes* ASAP1 (A0A2I3TKP4), *Macaca mulatta* ASAP1 ([A0A1D5Q4B3](http://www.uniprot.org/uniprot/A0A1D5Q4B3)), *Mus musculus* ASAP1 (Q9QWY8), *Rattus norvegicus* ASAP1 ([A0A0G2JX76](http://www.uniprot.org/uniprot/A0A0G2JX76)), *Canis lupus familiaris* ASAP1 (F1PW09), *Bos taurus* ASAP1 (A0A3Q1LJH0), *Sus scrofa* ASAP1 (A0A287B2W2), *Gallus gallus* ASAP1 (A0A1D5P9X9), *Xenopus tropicalis* ASAP1 (F6UK14), *Callorhinchus milii* ASAP1 ([A0A4W3JDV2](http://www.uniprot.org/uniprot/A0A4W3JDV2)), *Danio rerio* Asap1b (A0A2R8QMT3), *Danio rerio* Asap1a (A0A0R4IM47), *Esox lucius* ASAP1 (A0A3P8YQN7), *Danio rerio* Asap2a (Q5RG20), *Danio rerio* Asap2b (Q5TZC7), *Danio rerio* Asap3 (FIQYT8).

**Supplementary Figure 2** │ *asap1a or asap1b* morphants cannot exhibit altered susceptibility to *Mm* infection. (A, B, C, D) Representative fluorescence images and quantiﬁcation of bacterial burdens by ﬂuorescence pixel counts of control and *asap1a* morphants (A, B) or control and *asap1b* morphants (C, D) at 5 dpi with equivalent bacterial inocula by the yolk injection method (scale bar 500 μm); *P*-values calculated by the Mann-Whitney test. No statistical difference between *asap1a* or *asap1b* group and control group. *n* = 25 per group. (E, F, G, H) Representative fluorescence images and quantiﬁcation of bacterial burdens by ﬂuorescence pixel counts of control and *asap1a* morphants (E, F) or control and *asap1b* morphants (G, H) at 5 dpi with equivalent bacterial inocula by the intravenous injection method (scale bar 500 μm); *P*-values calculated by the Mann-Whitney test. No statistical difference between *asap1a* or *asap1b* group and control group. *n* = 20 per group. Each dot represents one larva. Mean ± SEM from three pooled independent experiments.

**Supplementary Figure 3** │ *asap1* morphants infected with *Mm* cannot induce a relatively higher mortality. (A) Survival of zebrafish mock (PBS)-injected or injected with *Mm* of 50 CFU (*n* = 50 per group) by the yolk injection method; (B) Survival of zebrafish mock (PBS)-injected or injected with *Mm* of 100 CFU (*n* = 50 per group) by the intravenous injection method. *, *P* < 0.05, no statistical difference between MOs-NC *Mm* group and MOs-*asap1 Mm* group. the survival curve analysis is Log-rank test. Data shown are representative of three independent experiments. *asap1* means *asap1a* and *asap1b.*

**Supplementary Figure 4 │** (**A, B**) Western blotting (**A**) and qRT-PCR (**B**) analysis of the expression efficiency of 5 dpf zebrafish by injecting with *asap1a and asap1b* mRNA in *asap1* morphants. *, *P* < 0.05, **, *P* < 0.01 versus control morphants using Student’s unpaired *t*-test. Data shown are representative of three independent experiments. The antibody was against both Asap1a and Asap1b. *asap1* means *asap1a* and *asap1b*.

**Supplementary Figure 5 │** *asap1* morphants do not affect the total macrophage number. (A) Representative light microscopy images of neutral red staining in control and *asap1* morphants at 3 dpf (scale bar 100 μm). (B) The neutral red positive cells posterior to the yolk tube in live uninfected morphants at 3 dpf were quantitated as the number of macrophages. No signiﬁcant differences in total macrophage number were shown using Student’s unpaired *t*-test. *n* (MOs-NC) = 25, *n* (MOs-*asap1*) = 30, each dot represents one larva. Mean ± SEM from two pooled independent experiments. *asap1* means *asap1a* and *asap1b*.
