# Supplementary figures and images for: Asap1 Affects the Susceptibility of Zebrafish to Mycobacterium by Regulating Macrophage Migration
Source: Front Cell Infect Microbiol. 2020 Oct 29;10:519503. doi: 10.3389/fcimb.2020.519503 (PMC7658321; doi:10.3389/fcimb.2020.519503)

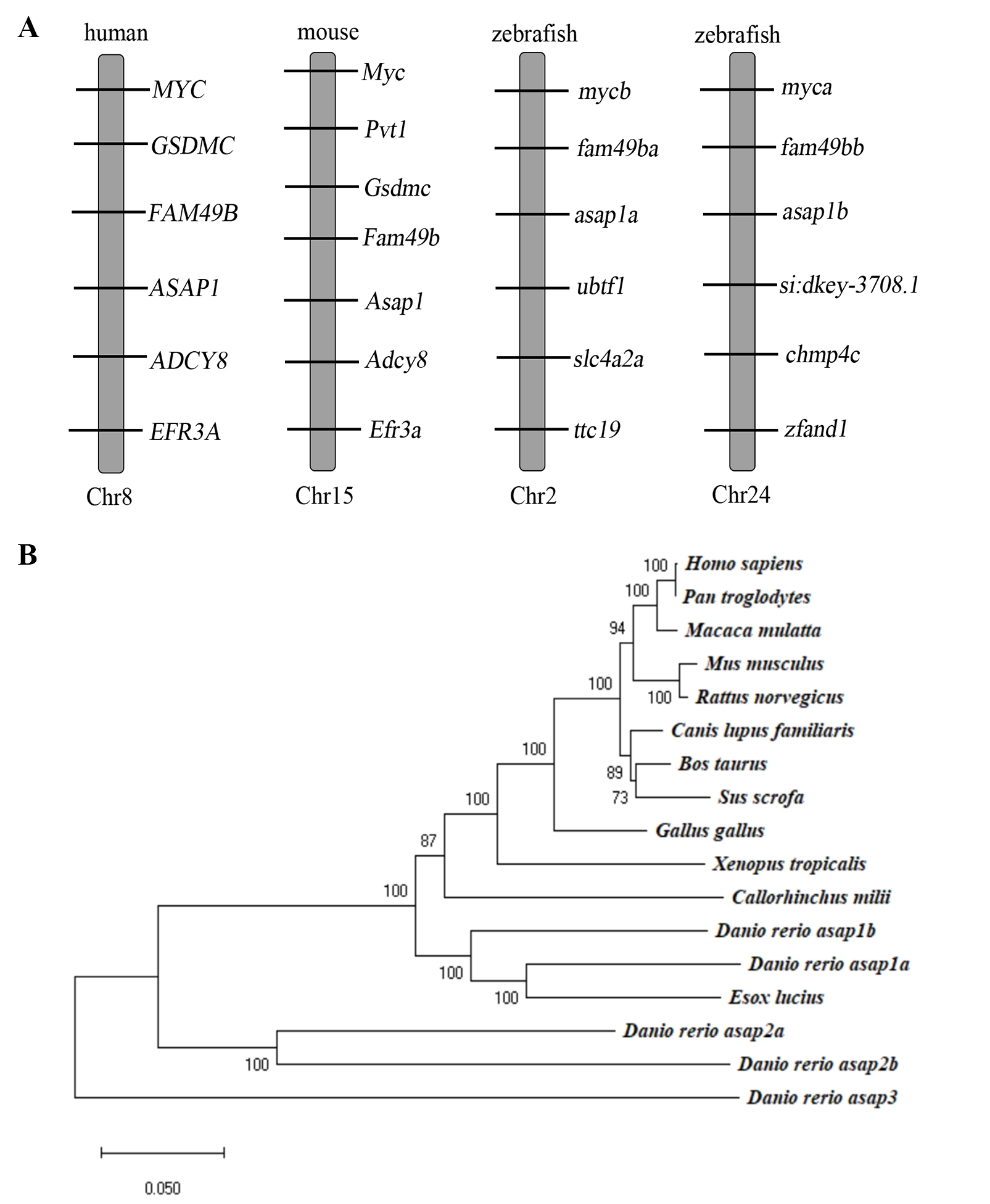

Supplement: Supplementary file 2 [file Data_Sheet_1.zip › Supplementary Figure S1.TIF]

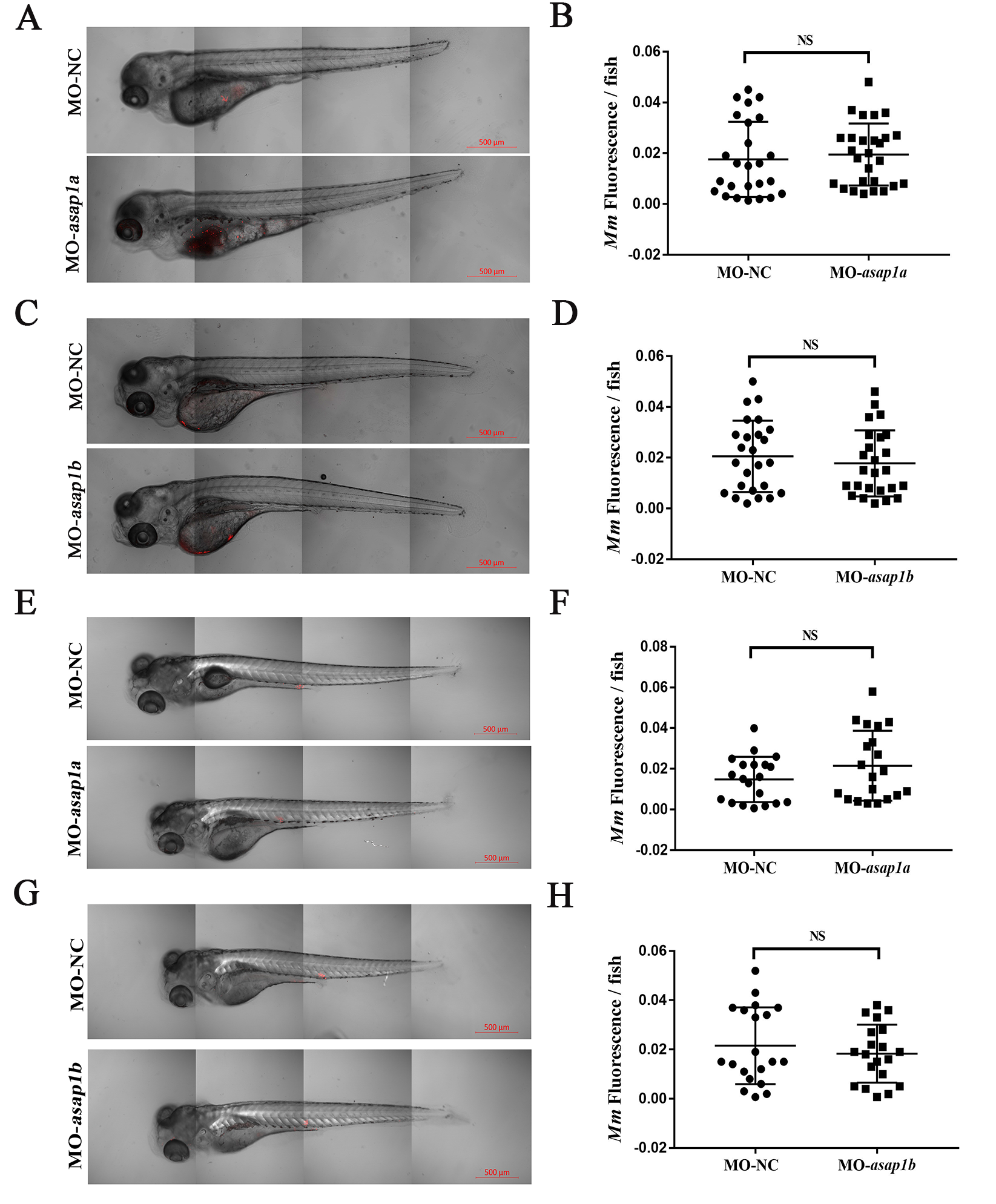

Supplement: Supplementary file 2 [file Data_Sheet_1.zip › Supplementary Figure S2.TIF]

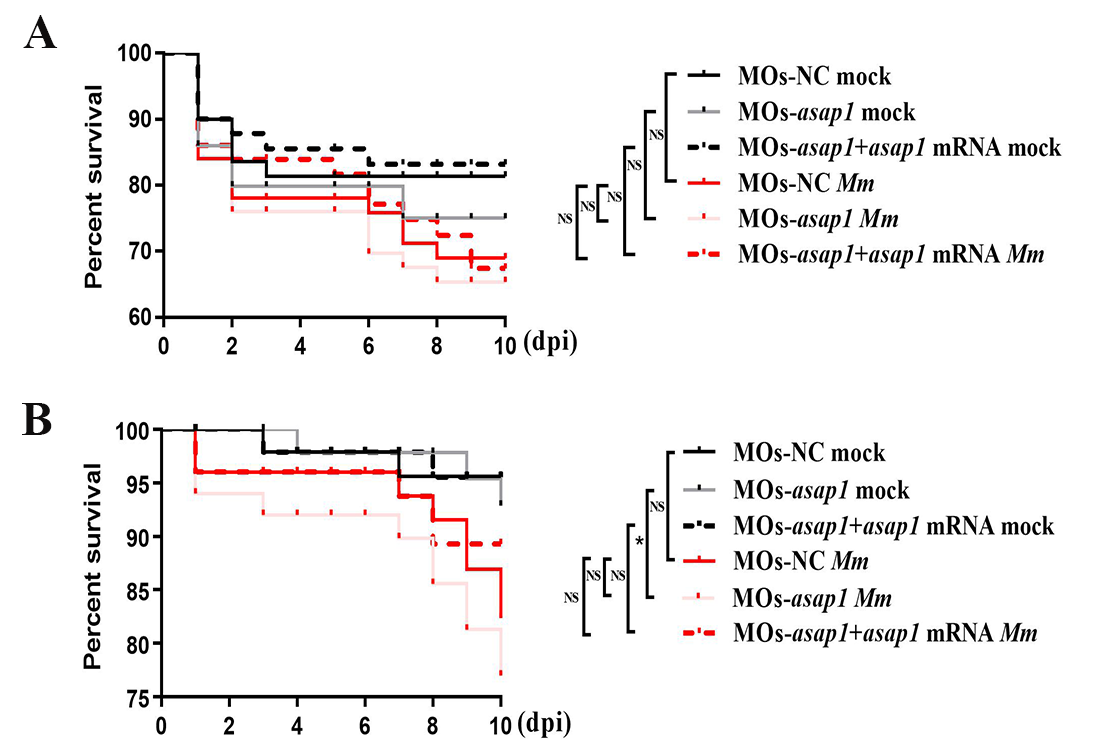

Supplement: Supplementary file 2 [file Data_Sheet_1.zip › Supplementary Figure S3.TIF]

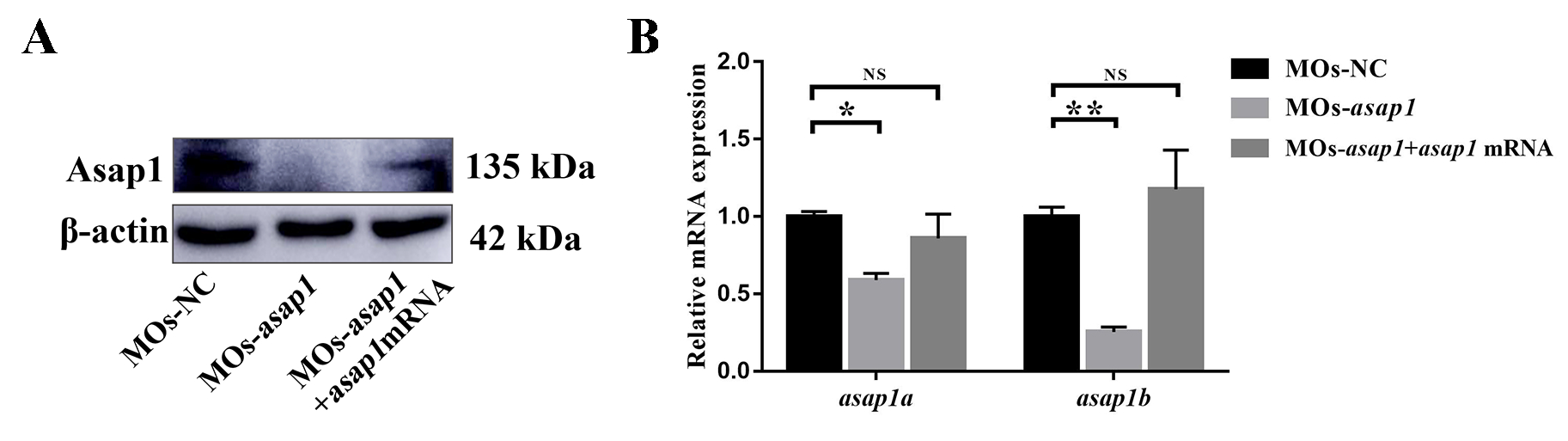

Supplement: Supplementary file 2 [file Data_Sheet_1.zip › Supplementary Figure S4.TIFF]

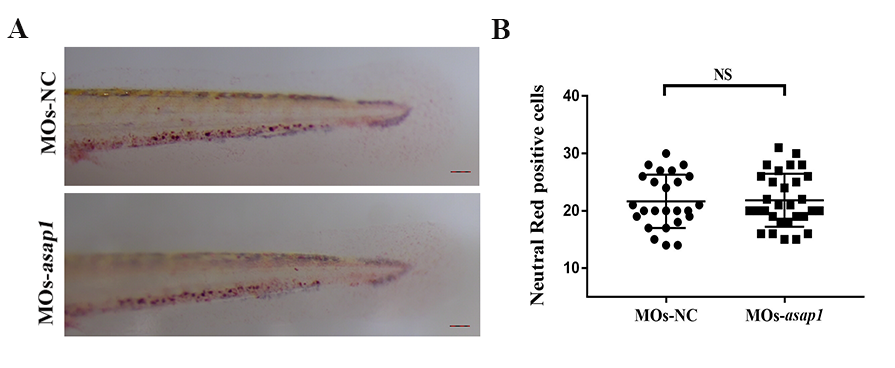

Supplement: Supplementary file 2 [file Data_Sheet_1.zip › Supplementary Figure S5.TIF]
